# Comparison of EPI DWI and STEAM DWI in Early Postoperative MRI Controls After Resection of Tumors of the Central Nervous System

**DOI:** 10.1007/s00062-023-01261-7

**Published:** 2023-02-02

**Authors:** Sebastian Johannes Müller, Eya Khadhraoui, Dirk Voit, Christian Heiner Riedel, Jens Frahm, Javier M. Romero, Marielle Ernst

**Affiliations:** 1 Department of Neuroradiology, University Medical Center Göttingen, Göttingen, Germany; 2 Max-Planck-Institute for Multidisciplinary Sciences, Göttingen, Germany; 3 Department of Radiology Division of Neuroradiology, Massachusetts General Hospital Harvard Medical School, Boston, MA USA; 4 Department of Neuroradiology, University Medicine Göttingen, Georg-August-University Göttingen, Robert-Koch-Str. 40, 37075 Göttingen, Germany

**Keywords:** STEAM, Early postoperative MRI, Susceptibility artifact, Infarction, Diffusion weighted imaging

## Abstract

**Purpose:**

Diffusion-weighted imaging (DWI) is important for differentiating residual tumor and subacute infarctions in early postoperative magnetic resonance imaging (MRI) of central nervous system (CNS) tumors. In cases of pneumocephalus and especially in the presence of intraventricular trapped air, conventional echo-planar imaging (EPI) DWI is distorted by susceptibility artifacts. The performance and robustness of a newly developed DWI sequence using the stimulated echo acquisition mode (STEAM) was evaluated in patients after neurosurgical operations with early postoperative MRI.

**Methods:**

We compared EPI and STEAM DWI of 43 patients who received 3‑Tesla MRI within 72 h after a neurosurgical operation between 1 October 2019 and 30 September 2021. We analyzed susceptibility artifacts originating from air and blood and whether these artifacts compromised the detection of ischemic changes after surgery. The DWI sequences were (i) visually rated and (ii) volumetrically analyzed.

**Results:**

In 28 of 43 patients, we found severe and diagnostically relevant artifacts in EPI DWI, but none in STEAM DWI. In these cases, in which artifacts were caused by intracranial air, they led to a worse detection of ischemic lesions and thus to a possible failed diagnosis or lack of judgment using EPI DWI. Additionally, volumetric analysis demonstrated a 14% smaller infarct volume detected with EPI DWI. No significant differences in visual rating and volumetric analysis were detected among the patients without severe artifacts.

**Conclusion:**

The newly developed version of STEAM DWI with highly undersampled radial encodings is superior to EPI DWI in patients with postoperative pneumocephalus.

**Supplementary Information:**

The online version of this article (10.1007/s00062-023-01261-7) contains supplementary material, which is available to authorized users.

## Introduction

In clinical practice diffusion-weighted imaging (DWI) based on echo-planar imaging (EPI) has a wide range of applications and is routinely used to detect cerebral infarctions [1]. The use of DWI with a single-shot stimulated echo acquisition mode (STEAM) sequence has been known for almost 30 years [2]. Although its inherent advantage is the lack of susceptibility artifacts, it is seldom applied because original implementations suffered from a limited signal-to-noise ratio.

Recent developments use a combination of a DW spin-echo module and a STEAM MRI readout with undersampled radial trajectories to provide an improved image quality [3, 4]. The new versions of STEAM DWI are now used routinely in our neuroradiologic institute in Göttingen. In a previous study [5] the capability of STEAM DWI for detecting subacute infarctions was evaluated and showed similar sensitivity and specificity as EPI DWI, but fewer artifacts.

Diffusion imaging is also one of the essential sequences in early postoperative MRI. The comparison of diffusion properties of tumor tissue before and after an operation in combination with T1-weighted, T2-weighted and contrast-enhanced imaging allows a better differentiation of residual tumor and subacute postoperative infarctions. A DWI is required as subacute infarctions may enhance [6] and mimic residual tumor. In cases of pneumocephalus and especially in the presence of intraventricular trapped air, EPI DWI is distorted by susceptibility artifacts. Therefore, the absence of such artifacts, as provided by STEAM DWI, could improve the quality of postoperative DWI. The purpose of the present study was to compare EPI DWI and STEAM DWI in patients after brain surgery, regarding sensitivity and specificity of infarct detection and infarct volume estimation, based on our relative gold standard of T2-weighted turbo spin echo (T2 TSE) sequence, 3 months after surgery.

## Methods

### Study Design

The MRIs of neurosurgical patients with early postoperative MRI were retrospectively analyzed. The study was ethically approved by the institutional review board (No. 32/05/21, Ethics Committee of the University of Göttingen).

### Subjects

We included all patients with early postoperative MRI, who were scanned between 1 October 2019 and 30 September 2021. Inclusion criteria were (i) a time interval up to 72 h after neurosurgical brain operation of brain tumors, (ii) a standard MRI protocol, as described in the next paragraph, and (iii) STEAM DWI.

The database search in our Radiology Information System (medavis RIS 4, medavis GmbH, Karlsruhe, Germany) with the search terms: “STEAM” and “postoperative” found 50 patients, 7 of whom were excluded because the interval between surgery and MRI was more than 3 days. Of the patients 43 (21 female) were included with an MRI scan performed within 72 h after surgery. Mean age at the time of MRI was 49 ± 22 years (mean ± standard deviation), range 4–81 years. The average time between end of the operation and the MRI was 37 ± 20 h (mean ± standard deviation, median 41 h).

The pathologies comprised 25 glioblastoma multiforme World Health Organization (WHO) grade IV, 3 high-grade astrocytomas WHO grade III, 10 low-grade gliomas WHO grade II, and 5 pilocytic astrocytomas WHO grade I.

### Image Acquisition

All MRIs were performed on the same 3‑Tesla MR scanner (MAGNETOM Prisma, Siemens, Munich, Germany). Our early postoperative MRI protocol included a high-resolution axial T2-weighted turbo spin echo (TSE) sequence with 2.5 mm slice thickness (echo time TE 108 ms, repetition time TR 3000 ms, in-plane resolution 0.5 × 0.5 mm), and a native and contrast-enhanced sagittal T1-weighted volume interpolated breathhold examination (VIBE) sequence with 1.0 mm slice thickness (TE 2 ms, TR 5 ms, in-plane resolution 1.0 × 1.0 mm). Additionally, an axial STEAM DWI sequence (TE 4.53 ms, TR 7.52 ms, 1 × 1 mm in-plane resolution, 3 mm slice thickness, voxel volume 3 mm^3^, 6 gradient directions, 144 s acquisition time, with DWI acquisition at b = 0 s/mm^2^ and b = 1000 s/mm^2^, apparent diffusion coefficient (ADC) map) was applied in tandem with a standard EPI DWI (Siemens 3scan_trace_p2, TE 67 ms, TR 3000 ms, 0.62 × 0.62 mm in-plane resolution, 5.2 mm slice thickness, voxel volume 2 mm^3^, 96 s acquisition time, with DWI acquisition at b = 0 s/mm^2^ and b = 1000 s/mm^2^, ADC map) in order to increase the detection rate for postoperative stroke in patients within 72 h after neurosurgery. For further details about the sequences, see Supplemental Table 1.

The STEAM DWI relies on a spin-echo diffusion module preceding a single-shot STEAM imaging technique with highly undersampled radial encodings. Iterative online image reconstruction is accomplished by nonlinear inversion with spatial regularization. The undersampling factor is 20, for further details see [3, 4].

The underlying technique of EPI DWI is conventionally a single-shot spin-echo echo planar imaging sequence with built-in diffusion gradients [7]. A 3-scan trace approach is used to account for the diffusion anisotropy. Image acquisitions are performed sequentially using 3 orthogonal diffusion gradient directions [7].

### Image Analysis

All patient data were pseudonymized and listed in a picture archiving and communication system (PACS) folder. Client software used was GE Centricity™ Universal Viewer (GE Healthcare, Chicago, IL, USA). Three neuroradiologists (rater 1, rater 2 and rater 3), with 2, 4 and 8 years of diagnostic MRI experience, respectively, independently evaluated the cases for the occurrence and severity of artifacts. The diagnostic accuracy regarding a residual tumor or a postoperative subacute infarction was assessed (i) with EPI DWI and without STEAM DWI and (ii) with STEAM DWI and without EPI DWI. Preoperative and postoperative T2-weighted and T1-weighted images with and without contrast agent were also available. A 4-week time interval was observed in order to minimize recall bias for the independent assessment of the two DWI sequences. The confidence in the imaging diagnosis of a residual tumor or a postoperative subacute infarction was indicated separately by each rater on a visual analog scale from 0 to 100%.

**Fig. 1:**
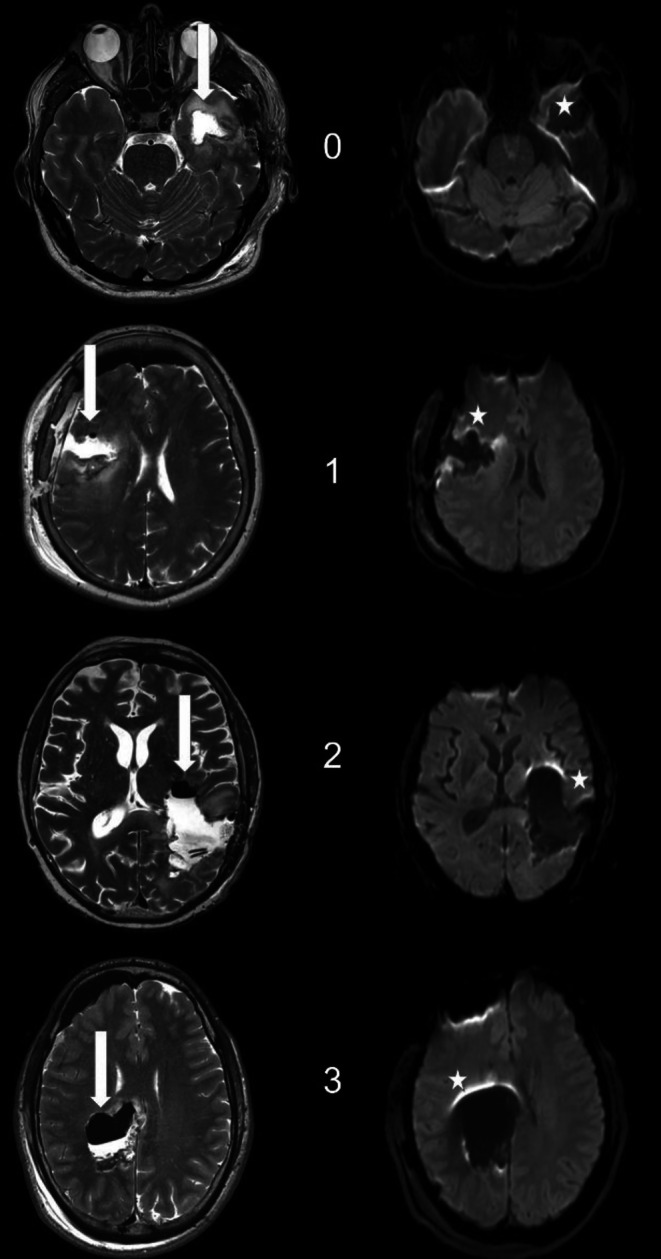
Susceptibility (air) artifacts rating scale (*left* T2-weighted turbo spin-echo images, *right* transversal echo-planar diffusion weighted images, EPI DWI, with b = 1000 s/mm^2^, *arrows* resection area with or without T2-hypointense air, *stars* associated area with or without air artifacts). Air artifacts rating (in the resection area): 0 no air artifacts, 1 small artifacts (e.g. small air bubbles) not affecting more than 5% of the brain tissue, 2 moderate artifacts affecting less than 25% of the neighboring brain tissue, 3 severe artifacts affecting more than 25% of the neighboring brain tissue

The T2-weighted TSE in combination with diffusion-weighted images with b = 1000 s/mm^2^ of (i) EPI and (ii) STEAM of every patient were rated for the presence and severity of air artifacts (AAR air artifacts rating; 0 no artifacts, 1 small artifacts, 2 moderate artifacts, 3 severe artifacts). The rating was done for 3 regions (frontal subdural, ventricular system and resection cavity). Figure 1 demonstrates the rating scale. For further analysis, MRI data were dichotomized for the presence of distorting artifacts in the resection cavity (0–1 without, 2–3 with influencing artifacts).

The resection cavity was rated for the presence and severity of blood artifacts as well.

We defined the transversal T2-weighted TSE sequence 3 months after surgery as our gold standard for the determination of sensitivity and specificity. All gold standard diagnoses were discussed and determined jointly. We considered every T2-hyperintense lesion or defect, (despite tumor growth) with a short axis diameter of at least 2 mm as an infarct (T2-weighted TSE transversal as described above). Connected lesions were counted as one.

### Confidence in Imaging Diagnosis

In addition to the visual determination of the infarction lesions and the indication whether they would suspect residual tumor, each radiologist had a subjective certainty of the diagnosis on a visual analog scale (0–10). An explicit image quality and sharpness rating of both sequences was not conducted.

### Semi-automated Volume Analysis

**Fig. 2:**
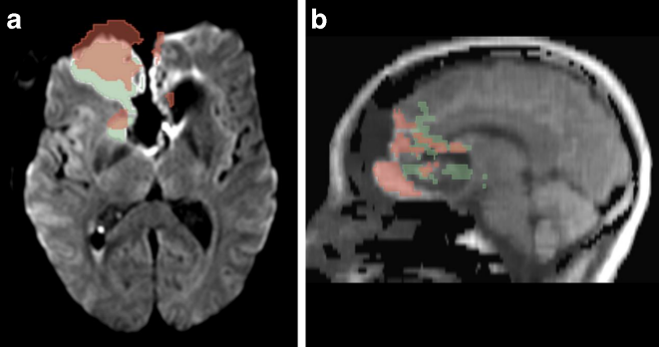
3D Slicer segmentation tool. Typical example of different segmentations of postoperative STEAM (*red*) and EPI (*green*) diffusion-weighted images with b = 1000 s/mm^2^. The segmentations are projected onto (**a**) an axial EPI DWI slice and **b** a reconstructed sagittal STEAM DWI slice of the same patient. The distortion of EPI DWI due to air artifacts in the frontal base of the skull becomes obvious

Two blinded raters (rater 1 and rater 2) separately marked all infarct areas using T2-weighted TSE transversal and either EPI DWI (with b = 1000 s/mm^2^ and ADC map) or STEAM DWI (with b = 1000 s/mm^2^ and STEAM ADC map) with the segmentation tool of 3D Slicer (http://3dslicer.org, version 4.11 Linux). Areas with an increased signal on b = 1000 s/mm^2^ imaging and a reduction of the corresponding ADC values were segmented in each slice, as demonstrated in Supplemental Fig. 1. All resection borders were counted (independent of thickness). Infarct volumes were calculated by 3D Slicer. The values of both raters were averaged for further analysis. An example of the segmentation tool is shown in Fig. 2.

### Statistical Analysis

The program Statistica, version 13 (TIBCO Software Inc., Palo Alto, CA, USA) was used for statistical analysis. *P*-values below 0.05 were defined as statistically significant. The detection rate, sensitivity and specificity of STEAM DWI to detect postoperative stroke as compared to EPI DWI was calculated for every individual rater as well as for all raters together. Shapiro-Wilk test [8] rejected the hypothesis of a normal distribution of the detection rates and measured volumes. Hence, the nonparametric Wilcoxon matched pairs [9] test was used for the comparison of observations and volume data.

Fleiss’ kappa [10] was calculated to determine the overall raw agreement between the three neuroradiologists.

## Results

### Detection Rate, Sensitivity and Specificity

Postoperative diffusion-restricted lesions (diameter > 3 mm) were detected in 43 of 43 patients. On average 2.2 ± 1.4 (mean ± standard deviation, rater 1: 2.1 ± 1.3, rater 2: 2.2 ± 1.4, rater 3: 2.3 ± 1.4) diffusion-restricted lesions were detected per patient using EPI DWI and 2.9 ± 1.3 (rater 1: 2.8 ± 1.2, rater 2: 2.9 ± 1.4, rater 3: 3.0 ± 1.4) lesions using STEAM DWI. An illustration of the frequency distribution is provided in Supplemental Fig. 2.

In patients with air artifacts (AAR > 1), the mean count of diffusion-restricted lesions was 2.0 ± 1.3 (EPI DWI), and 2.8 ± 1.2 (STEAM DWI).

Shapiro-Wilk test showed non-normally distributed data. Wilcoxon matched pairs test samples showed a significant difference between methods (*p* < 0.01), with a higher number of detected lesions using STEAM DWI.

In 26 patients a 3-month follow-up MRI was available (gold standard). For these patients, the overall detection rate (for infarct lesions) of EPI DWI was 74% (63/85) compared to 100% of STEAM DWI. Subacute infarct lesions were completely missed by EPI DWI in two patients (sensitivity: EPI DWI 92%, STEAM DWI 100%). Specificity was 100% for both sequences.

A postoperative irradiation following the Stupp protocol [11] was already started in 16 patients at the time of follow-up.

### Confidence in Imaging Diagnosis

The mean diagnostic confidence (on visual analog scale 0–10, three neuroradiologists) for the detection of infarct lesions was 64 ± 28% for EPI DWI (significant difference, paired t‑test, *p* < 0.001), and 96 ± 17% for STEAM DWI and for residual tumors 89 ± 23% and 95 ± 14%, respectively (no significant difference, paired t‑test, *p* = 0.099).

### Interrater Reliability

Overall Fleiss’ kappa for the counting of infarct lesions (three raters) was 0.60 (moderate agreement) for EPI DWI and 0.57 (moderate agreement) for STEAM DWI. We noticed more counted lesions in STEAM than in EPI DWI for each rater. The interrater differences were not primarily based on different visual detections, but on the fact that two or three directly adjacent lesions were counted differently by the raters, either as one contiguous lesion or separately. Logically, Fleiss’ kappa for the difference of counted infarct lesions in EPI and STEAM DWI showed a better, substantial agreement of 0.70 (3 raters). As this quantitative assessment did not seem sufficient, we also carried out a volumetric analysis.

### Volumetric Analysis

In 43 of 43 patients, measurable infarcts were detected by both DWI sequences. The mean infarct volume per patient was 5.8 ± 5.5 cm^3^ (mean ± standard deviation, median 4.2 cm^3^, minimum 0.1 cm^3^, maximum 25 cm^3^) using STEAM DWI with b = 1000 s/mm^2^ and ADC and 5.1 ± 5.5 cm^3^ (median 4.0 cm^3^, minimum 0 mm^3^, maximum 25 cm^3^) using EPI DWI with b = 1000 s/mm^2^ and ADC.

In cases of no severe air (*n* = 15) in the resection cavity, both DWI showed similar results (STEAM 4.2 ± 2.7 cm^3^ vs. EPI DWI 4.0 ± 2.4 cm^3^). For the 28 cases with air artifacts, an average volume of 6.6 ± 5.9 cm^3^ was detected using STEAM DWI, and 5.8 ± 5.8 cm^3^ using EPI DWI. Table 1 displays the measured volumes.

**Table 1:** Measured infarct volumes in diffusion weighted images of stimulated echo acquisition mode (STEAM) and echo-planar imaging (EPI) sequences using 3D Slicer software (group 1 all measured patients, group 2 measured patients with severe air artifact in the resection cavity; group 3 measured patients without severe air artifact in the resection cavity)

	Unit	Group 1		Group 2		Group 3	
		EPI	STEAM	EPI	STEAM	EPI	STEAM
*n*	–	43	43	28	28	15	15
*Mean*	cm^3^	5.14	5.76	5.77	6.58	3.95	4.22
*Standard deviation*	cm^3^	5.52	5.48	5.85	5.94	2.41	2.66
*Median*	cm^3^	4.07	4.26	4.21	5.31	3.62	3.79
*Minimum*	cm^3^	0.00	0.12	0.00	0.36	0.10	0.12
*Maximum*	cm^3^	25.98	25.72	25.98	25.73	10.49	13.01

**Fig. 3:**
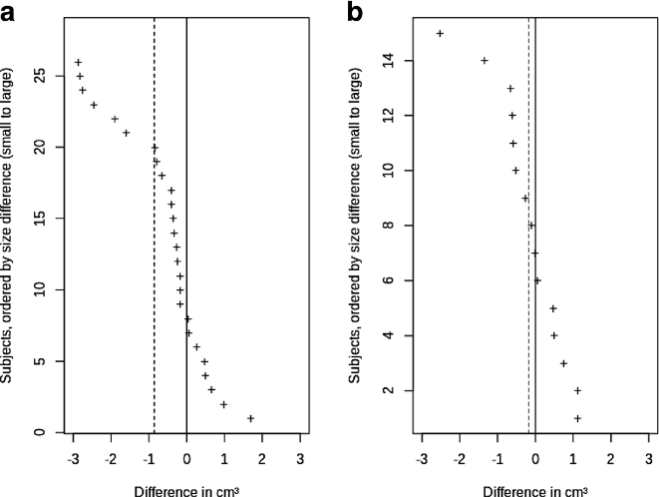
Differences of the mean measured postoperative ischemic volumes in cm^3^ using STEAM and EPI DWI. **a** Patients with air artifacts, **b** without air artifacts. The shift in the left chart indicates a systematic difference as a result of the missing susceptibility artifacts in STEAM DWI. *Dotted line* mean difference volume (EPI minus STEAM DWI)

Wilcoxon matched pairs test showed significant (*p* < 0.02) differences between both (EPI and STEAM DWI) volumes in patients with severe air artifacts in the resection cavity, but not in patients without such artifacts (*p* > 0.5). A graphic illustration of the results is given in Fig. 3.

### Air and Blood Artifacts

**Fig. 4:**
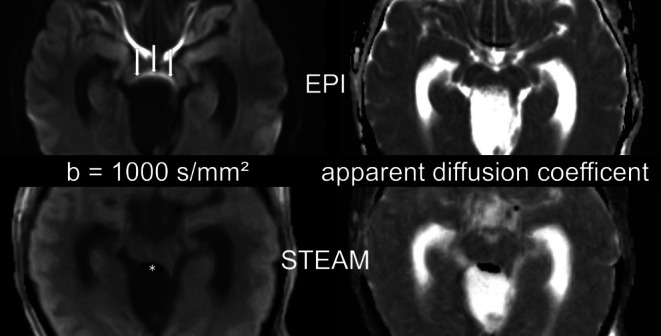
23-year-old female. Postoperative b = 1000 s/mm^2^ images (*left*) and apparent diffusion coefficient (ADC) maps (*right*) obtained by EPI DWI (*top*) and STEAM DWI (*bottom*) 40 h after resection of an infratentorial arachnoid cyst with compression of the brain stem. In EPI DWI only hyperintensities (*arrows*) of the dorsal mesencephalon caused by susceptibility artifacts were noticed. An infarction could not be excluded. In STEAM DWI air (*asterisk*) and the compression of the brain stem can be detected. An infarction of the mesencephalon could be excluded

Intracranial air was noticed in every patient. EPI DWI presented with corresponding artifacts in every case. In more detail, air was seen as frontal pneumocephalus in 39 patients, in the resection cavity in 33 cases and in the ventricular system in 16 cases. An illustrative case showing the benefits of STEAM DWI is demonstrated in Fig. 4.

More specifically, the air in the resection cavity was in the form of a small microbubble (AAR 1) in 5 cases. In 28 patients (24 patients AAR 2, 4 patients AAR 3), EPI DWI was distorted by air artifacts in the resection cavity, especially in very early postoperative controls (< 24 h). Table 2 illustrates the distribution of air and blood artifacts.

**Table 2:** Distribution of air and blood artifact in the resection cavity using EPI DWI (echo-planar imaging diffusion weighted imaging)

Air artifacts (AAR)	n	Significant distorting?	m
*0*	10	No	15
*1*	5		
*2*	24	Yes	28
*3*	4		
*Blood artifacts*	*n*	*Significant distorting?*	*m*
*None*	26	No	43
*Minor*	8		
*Moderate*	9		
*Severe*	0	Yes	0

*n, m* number of patients, *AAR* air artifact rating

**Fig. 5:**
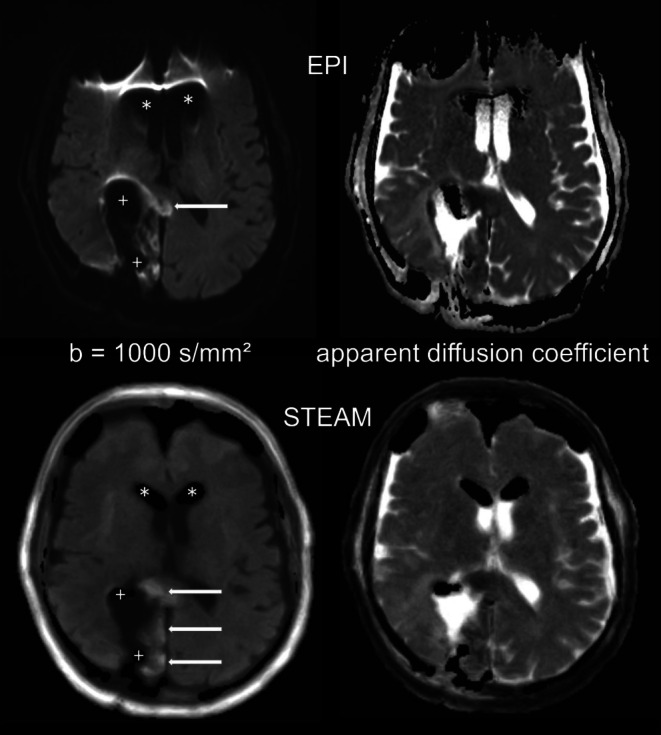
81-year-old patient. Postoperative diffusion weighted images with b = 1000 s/mm^2^ (*left*) and apparent diffusion coefficient (ADC) maps (*right*) obtained by EPI DWI (*top*) and STEAM DWI (*bottom*) 42 h after resection of a glioblastoma of the right parieto-occipital region. The patient presented with trapped air in the ventricular system (*asterisk* ) and the resection cavity (+) and with an infarction of the occipital splenium and the parasagittal occipital lobe (*arrows*)

The mean sum (per rater) of detected infarct lesions using EPI DWI was 89, while an average total of 120 lesions was identified via STEAM DWI, as detailed shown in Table 3. It is not surprising that more lesions were found even in the group without air artifacts, as there were of course susceptibility artifacts due to blood deposits in this group as well. Figure 5 shows an example of a resected high-grade glioma.

**Table 3:** Counted infarct lesions using echo-planar imaging (EPI) and stimulated echo acquisition mode (STEAM) diffusion weighted images (DWI). Group 1 contains all measured patients, group 2 is the subgroup of patients with and group 3 the subgroup without severe air artifact in the resection cavity

	Group 1 *n* = 43		Group 2 *n* = 28		Group 3 *n* = 15	
DWI	EPI	STEAM	EPI	STEAM	EPI	STEAM
*Count (rater 1)*	73	103	47	70	26	33
*Count (rater 2)*	96	127	57	82	39	45
*Count (rater 3)*	98	131	61	88	37	43
*Mean (count raters 1–3)*	89	120	55	80	34	40
*Mean (rater 1)*	1.70	2.40	1.68	2.50	1.73	2.20
*Mean (rater 2)*	2.23	2.95	2.04	2.93	2.60	3.00
*Mean (rater 1)*	2.28	3.05	2.18	3.14	2.47	2.87
*Mean (mean raters 1–3)*	2.07	2.80	1.96	2.86	2.27	2.69

In 17 patients with a 3-month follow-up and moderate air artifacts (AAR > 1), EPI DWI detected 36 of 58 lesions (sensitivity 62%, STEAM DWI 100%) compared to the gold standard (3-month follow-up MRI, T2-weighted TSE). In patients with less air artifacts both sequences had a sensitivity of 100% (3-month follow-up MRI, AAR < 2, *n* = 9, 27/27 lesions).

Blood artifacts were noticed in 17 patients using EPI DWI, but did not appear severe (9 cases moderate, 8 cases minor). Blood or air artifacts were not noticed using STEAM DWI. In 14 patients, neither severe air nor blood artifacts were noticed in the operation area using EPI DWI. In these cases, postoperative infarctions were equally detected with both sequences.

## Discussion

Our study of 43 patients demonstrates that the new version of the STEAM DWI technique in early postoperative MRI offers additional information for a more reliable diagnosis of ischemic lesions and thus better differentiation of residual tumor and postoperative infarction. The fragility of the EPI DWI to susceptibility artifacts results from the readout of data from multiple gradient echoes, all of which are sensitive to susceptibility differences [7]. To prevent this, the STEAM DWI uses a sequence of stimulated refocused echoes to compensate for such susceptibility artifacts [4].

Hence, in cases where air artifacts completely distort EPI DWI, a more confident diagnosis using STEAM DWI is possible. Previous studies [5, 12] found STEAM detection rates in stroke patients to be slightly worse than those for EPI DWI, mainly due to a lower signal-to-noise ratio, resulting in poorer detection of microembolisms. The present work reveals a superiority of STEAM DWI in the detection of acute or subacute strokes in patients with postoperative air artifacts using a new version of this sequence. The detection rate of ischemic lesions was significantly higher in cases with such susceptibility artifacts compared to EPI DWI. Moreover, despite only moderate interrater reliability, the volumetric results promise excellent applicability in clinical practice, i.e., for both early postoperative and intraoperative MRI.

For the first time, STEAM DWI allows reliable exclusion of ischemic lesions, even with large amounts of intracranial air and blood. For this reason, STEAM DWI should be included in a standard set-up for postoperative imaging with intracranial air.

The extent to which better detection of such lesions influences treatment and prognosis is not yet clear. It is well known that preoperative DWI with ADC maps is a very useful tool and can even serve as a prognostic biomarker [13]. Especially in patients with a glioblastoma, the existence of perifocal DWI hyperintensities is a very poor prognostic factor [14], i.e., the complete resection of such diffusion-restricted tumor tissue is particularly important. A Swiss study showed the limitations of DWI and that intraoperative infarctions in particular can be overlooked or underestimated [15]. There are different study results on the existence and prognostic value of postoperative infarcts. While a study from Japan [16] classified this as prognostically favorable, presumably because micro-invasive tumor cells can also die off in the postoperatively infarcted tissue, the opposite is the case in a study from Munich [17], where the authors assumed that hypoxia may promote more aggressive tumor growth. Postoperative infarcts are also influenced by the technique of the neurosurgeon as well as a possible preference for excessive hemostasis using the bipolar electrocoagulation and thus vary greatly from clinic to clinic.

Early postoperative MRI plays an important role in assessing the prognosis of a patient with a high-grade brain tumor [18], especially in cases of glioblastoma [19] and may lead to additional surgery. Precise determination and differentiation between postoperative infarctions and residual tumor are essential [20]. Whereas a thin linear enhancement pattern is more common in early postoperative MRI and can usually be neglected as necrotic tissue damage due to resection, a thick linear enhancement is suggestive of residual tumor [21]; however, this contrast enhancement could also be a subacute stroke [6] masquerading as residual tumor. Especially in recurrent glioblastoma, such ischemic changes occur more frequently [22]. The most important prognostic factor remains the extent of resection [21]. As one of the main disadvantages of EPI-DWI is image distortion due to artifacts caused by air or blood [23], the extent of resection can be better estimated using STEAM DWI, which offers undistorted anatomy.

### Influence on the Diagnosis

In general, the contribution of DWI alone to the quality of postoperative imaging is multifactorial, subjective and hard to measure. Of particular importance, however, is the advantage of being able to safely rule out infarction in symptomatic patients, as shown in Fig. 5.

**Fig. 6:**
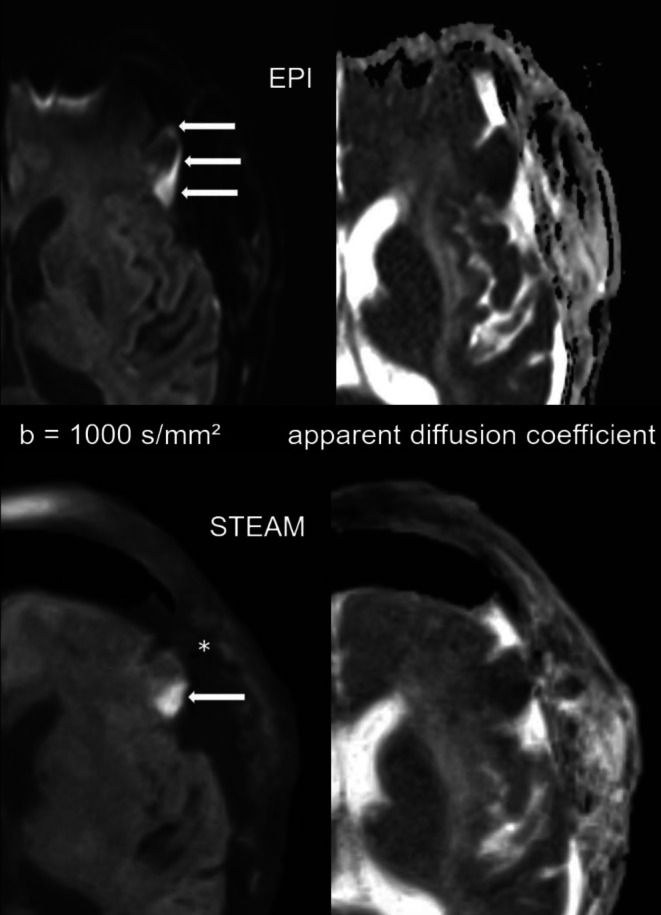
77-year-old patient. Postoperative diffusion-weighted images with b = 1000 s/mm^2^ (*left*) and apparent diffusion coefficient maps (*right*) obtained by EPI DWI (*top*) and STEAM DWI (*bottom*) 71 h after resection of a left temporal astrocytoma. In EPI DWI the border between infarct area (*arrows*), cerebrospinal fluid and air is not clear due to geometric distortion, whereas in STEAM DWI the infarct area (*arrow*) is sharply outlined and even small air bubbles (*asterisk*) can be detected

In 3 of 25 patients with glioblastoma, we detected T2-hyperintense, contrast-enhanced tissue and correlating distorting susceptibility artifacts on EPI DWI. In these cases, STEAM DWI helped to reliably differentiate between a contrast-enhancing residual tumor and a subacute infarct (see Fig. 6 for an example).

In general, we expect an even higher benefit of STEAM DWI in early postoperative MRI for low-grade gliomas, which could not be finally shown in this study due to the small number of cases and only minor air inclusions in these patients.

### Air in the Ventricular System

In the cases with intraventricular air STEAM DWI showed better anatomic visualization of the adjacent brain areas and postoperative infarctions. In these cases it was possible to match infarct location and contrast enhancement to reliably reveal the residual tumor.

### Study Limitations

The only moderate interrater agreement is not surprising, as the detection and counting of ischemic changes after surgery is a complex task that requires three-dimensional imagination of contiguous regions and is difficult to objectify. Different voxel size, slice thickness and volume of the two DWIs may influence the measured volumes by partial volume effects [24]. If this were the case, the volumes of patients without air artifacts should also differ, but they do not.

A longitudinal follow-up of several months would increase the confidence of the diagnosis in the case of residual tumors. At the time of this evaluation, only 26 follow-up scans (gold standard) had been performed. As these cases already showed significant differences (EPI 74% vs. STEAM 100% sensitivity, both 100% specificity) and a clear superiority, we finished the analyses. An explicit image quality and sharpness rating of both sequences was not carried out because we suspected a strong correlation with diagnostic confidence.

Unfortunately, we could not test the sequence in intraoperative MRI, as this is not common practice in our hospital. Additional information can be expected in this set-up as well.

This study served as a pilot project to gain first experience with STEAM DWI in postoperative MRI. Further studies with larger sample size are required to corroborate these findings. Different classes of pathologies could also be evaluated, e.g., intracranial hematomas or aneurysms.

## Conclusion

In patients with pneumocephalus the new version of the STEAM DWI with highly undersampled radial encodings is superior to the standard EPI DWI for the detection of postoperative infarct lesions. It should be included in clinical protocols to distinguish between residual tumor and extension of postoperative infarction.

## Supplementary Information

**Supplemental Table 1**—Parameters of magnet resonance imaging (MRI) sequences on 3‑Tesla used in the postoperative protocol. *TE* echo time, *TR* repetition time, *DWI* diffusion-weighted imaging, *STEAM* stimulated echo acquisition mode, *EPI* echo planar imaging, *TSE* turbo spin echo, *VIBE* volumetric interpolated breath-hold examination.

**Supplemental Fig. 1**—Screen shot of 3D slicer software. Example segmentation of four postoperative ischemic areas (red, blue, yellow and green) in a slice. After half-automated segmentation in every slice (slice thickness 3 mm, approx. 50 slices), volumes were calculated automatically by the software. *Left* STEAM diffusion weighted images with b = 1000 s/mm^2^; *right*: STEAM ADC. *STEAM* stimulated echo acquisition mode, *ADC* apparent diffusion coefficient.

**Supplemental Fig. 2**—Frequency distribution of the rated lesions per patient. *STEAM* stimulated echo acquisition mode, *EPI* echo planar imaging, *R1, R2, R3* rater 1, rater 2 and rater 3 are the three neuroradiologists.
