# Supplementary material for: Comparison of EPI DWI and STEAM DWI in Early Postoperative MRI Controls After Resection of Tumors of the Central Nervous System
Source: Clin Neuroradiol. 2023 Feb 2;33(3):677–85. doi: 10.1007/s00062-023-01261-7 (PMC10449950; doi:10.1007/s00062-023-01261-7)
Supplement: Supplementary file 1 — Supplemental Table 1—Parameters of magnet resonance imaging (MRI) sequences on 3‑Tesla used in the postoperative protocol. TE echo time, TR repetition time, DWI diffusion-weighted imaging, STEAM stimulated echo acquisition mode, EPI echo planar imaging, TSE turbo spin echo, VIBE volumetric interpolated breath-hold examination. [file 62_2023_1261_MOESM1_ESM.docx]

**Comparison of EPI DWI and STEAM DWI in early postoperative MRI controls after resection of tumors of the Central Nervous System**

**Supplemental Table 1** – Parameters of MRI (magnet resonance imaging) sequences on 3-Tesla used in the postoperative protocol.

| **Parameter/**  **Sequence** | **STEAM DWI** | **EPI DWI** | **T2 TSE** | **T1 VIBE** |
| --- | --- | --- | --- | --- |
| **Acquisition time** | 144 s | 96 s | 254 s | 118 s |
| **In-plane-resolution** | 1 x 1 mm | 0.6 x 0.6 mm | 0.5 x 0.5 mm | 1 x 1 mm |
| **Slice thickness** | 3 mm | 5.2 mm | 2.5 mm | 1.0 mm |
| **TE** | 4.53 ms | 67 ms | 108 ms | 2.24 ms |
| **TR** | 7.52 ms | 3000 ms | 3000 ms | 5 ms |
| **Orientation** | transversal | transversal | transversal | sagittal |

Legend: TE – echo time, TR – repetition time, DWI – diffusion weighted imaging, STEAM - stimulated echo acquisition mode, EPI – echo planar imaging, TSE – turbo spin echo, VIBE - Volumetric Interpolated Breath-hold Examination.
