# Supplementary material for: Comparison of EPI DWI and STEAM DWI in Early Postoperative MRI Controls After Resection of Tumors of the Central Nervous System
Source: Clin Neuroradiol. 2023 Feb 2;33(3):677–85. doi: 10.1007/s00062-023-01261-7 (PMC10449950; doi:10.1007/s00062-023-01261-7)
Supplement: Supplementary file 2 — Supplemental Fig. 1—Screen shot of 3D slicer software. Example segmentation of four postoperative ischemic areas (red, blue, yellow and green) in a slice. After half-automated segmentation in every slice (slice thickness 3 mm, approx. 50 slices), volumes were calculated automatically by the software. Left STEAM diffusion weighted images with b = 1000 s/mm2; right: STEAM ADC. STEAM stimulated echo acquisition mode, ADC apparent diffusion coefficient. [file 62_2023_1261_MOESM2_ESM.docx]

**Comparison of EPI DWI and STEAM DWI in early postoperative MRI controls after resection of tumors of the Central Nervous System**

**Supplemental Figure 1** – Screen shot of 3D slicer software. Example segmentation of four postoperative ischemic areas (red, blue, yellow and green) in a slice. After half-automated segmentation in every slice (slice thickness 3 mm, approx. 50 slices), volumes were calculated automatically by the software. Left: STEAM diffusion weighted images with b = 1000 s/mm²; right: STEAM ADC.


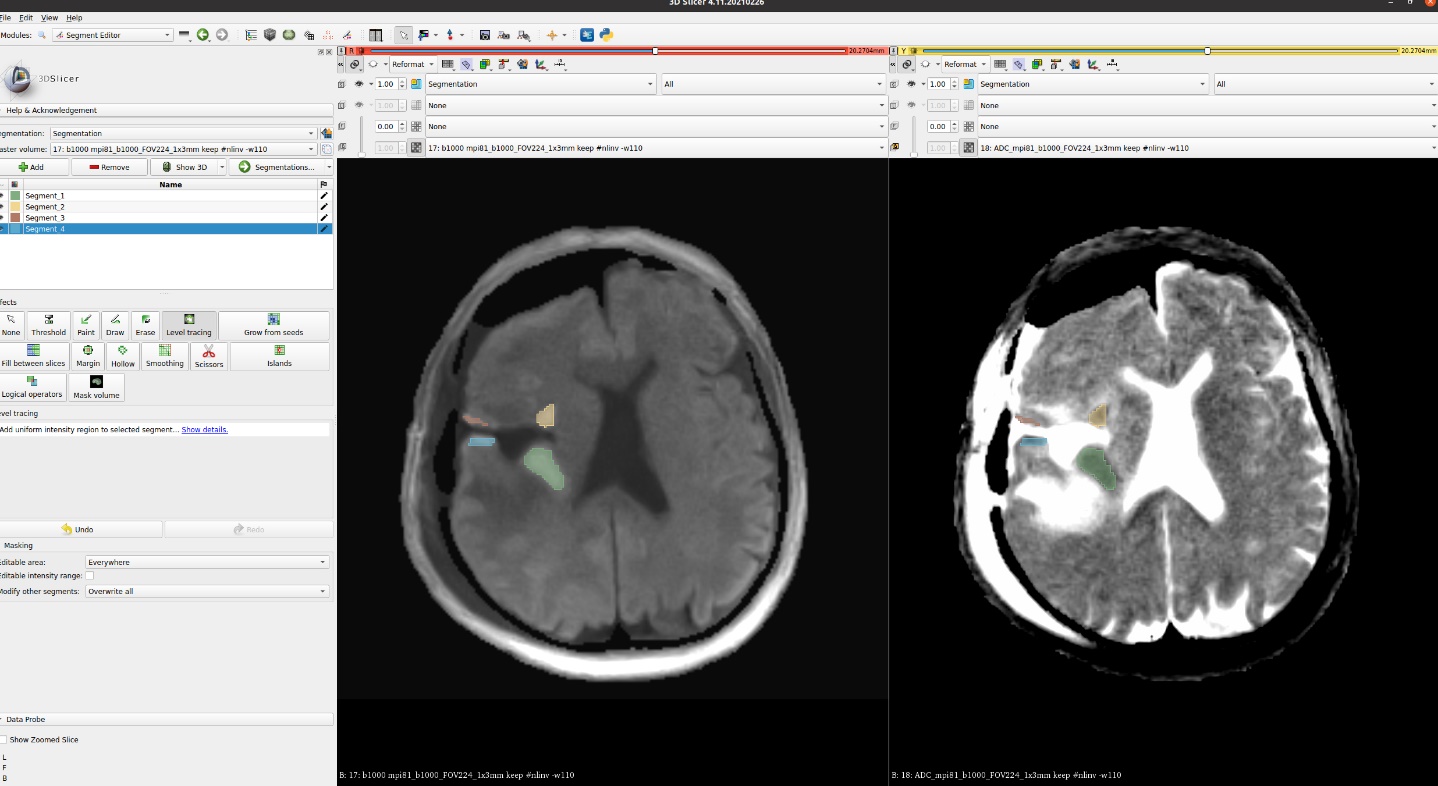


Legend: STEAM - stimulated echo acquisition mode, ADC apparent diffusion coefficient.
