# Supplementary material for: Comparison of EPI DWI and STEAM DWI in Early Postoperative MRI Controls After Resection of Tumors of the Central Nervous System
Source: Clin Neuroradiol. 2023 Feb 2;33(3):677–85. doi: 10.1007/s00062-023-01261-7 (PMC10449950; doi:10.1007/s00062-023-01261-7)
Supplement: Supplementary file 3 — Supplemental Fig. 2—Frequency distribution of the rated lesions per patient. STEAM stimulated echo acquisition mode, EPI echo planar imaging, R1, R2, R3 rater 1, rater 2 and rater 3 are the three neuroradiologists. [file 62_2023_1261_MOESM3_ESM.docx]

**Comparison of EPI DWI and STEAM DWI in early postoperative MRI controls after resection of tumors of the Central Nervous System**

**Supplemental Figure 2** – Frequency distribution of the rated lesions per patient.


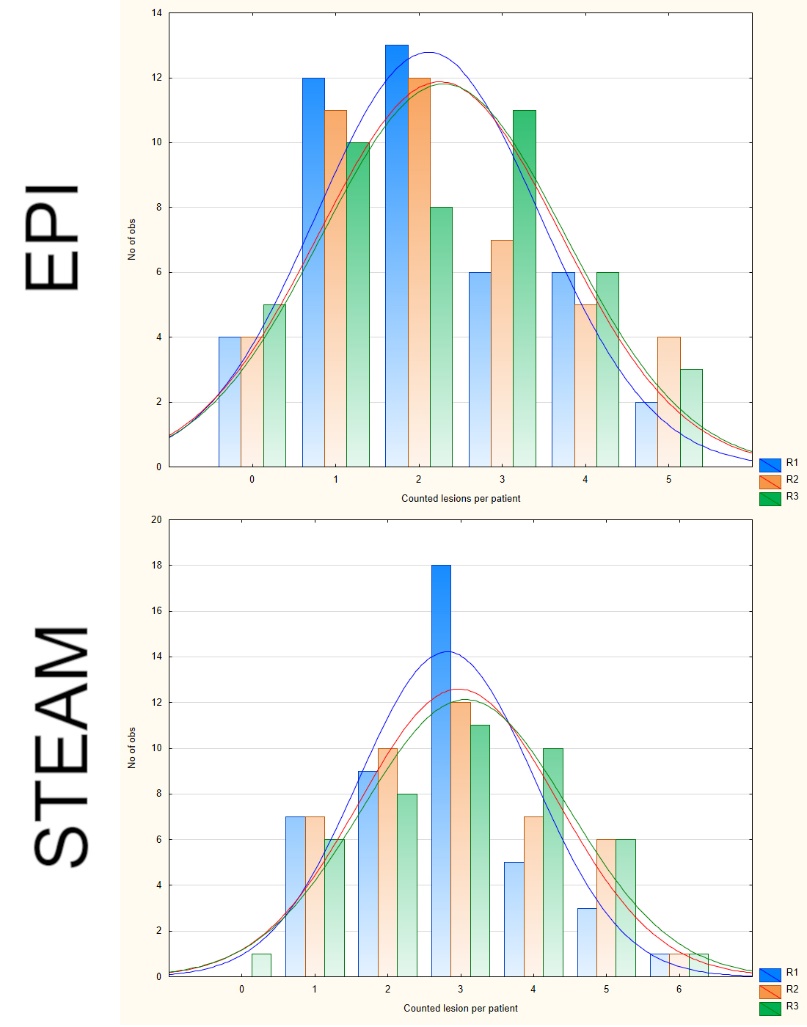


Legend: STEAM - stimulated echo acquisition mode, EPI – echo planar imaging. R1, R2, R3 – rater 1, rater 2 and rater 3 are the three neuroradiologists.
